# Babesiosis Occurrence among the Elderly in the United States, as Recorded in Large Medicare Databases during 2006–2013

**DOI:** 10.1371/journal.pone.0140332

**Published:** 2015-10-15

**Authors:** Mikhail Menis, Richard A. Forshee, Sanjai Kumar, Stephen McKean, Rob Warnock, Hector S. Izurieta, Rahul Gondalia, Chris Johnson, Paul D. Mintz, Mark O. Walderhaug, Christopher M. Worrall, Jeffrey A. Kelman, Steven A. Anderson

**Affiliations:** 1 Food and Drug Administration, Silver Spring, Maryland, United States of America; 2 Acumen LLC, Burlingame, California, United States of America; 3 Centers for Medicare & Medicaid Services, Baltimore, Maryland, United States of America; University of Kentucky College of Medicine, UNITED STATES

## Abstract

**Background:**

Human babesiosis, caused by intraerythrocytic protozoan parasites, can be an asymptomatic or mild-to-severe disease that may be fatal. The study objective was to assess babesiosis occurrence among the U.S. elderly Medicare beneficiaries, ages 65 and older, during 2006–2013.

**Methods:**

Our retrospective claims-based study utilized large Medicare administrative databases. Babesiosis occurrence was ascertained by recorded ICD-9-CM diagnosis code. The study assessed babesiosis occurrence rates (per 100,000 elderly Medicare beneficiaries) overall and by year, age, gender, race, state of residence, and diagnosis months.

**Results:**

A total of 10,305 elderly Medicare beneficiaries had a recorded babesiosis diagnosis during the eight-year study period, for an overall rate of about 5 per 100,000 persons. Study results showed a significant increase in babesiosis occurrence over time (p<0.05), with the largest number of cases recorded in 2013 (N = 1,848) and the highest rates (per 100,000) in five Northeastern states: Connecticut (46), Massachusetts (45), Rhode Island (42), New York (27), and New Jersey (14). About 75% of all cases were diagnosed from May through October. Babesiosis occurrence was significantly higher among males vs. females and whites vs. non-whites.

**Conclusion:**

Our study reveals increasing babesiosis occurrence among the U.S. elderly during 2006–2013, with highest rates in the babesiosis-endemic states. The study also shows variation in babesiosis occurrence by age, gender, race, state of residence, and diagnosis months. Overall, our study highlights the importance of large administrative databases in assessing the occurrence of emerging infections in the United States.

## Introduction

Human babesiosis is a zoonotic disease caused by intraerythrocytic protozoan parasites of the genus *Babesia*. In the United States, *Babesia microti* is the primary etiologic agent of human babesiosis, which is usually transmitted via the bite of *Ixodes scapularis*, the principal tick vector [1–6]. Human *B*. *microti* infections are considered endemic in Northeastern states of Connecticut, Rhode Island, Massachusetts, New York, and New Jersey as well as in Midwestern states of Minnesota and Wisconsin, and the geographic range of the disease is expanding [2–10]. While human babesiosis infections are both regional and seasonal, with the peak transmission occurring during the summer months and mostly in endemic states, transfusion-associated cases may occur year round and anywhere in the country due to prolonged parasitemia in some donors, donor travel, and shipments of blood components throughout the country [1–5,9–14]. In younger persons, *B*. *microti* infection is likely to be asymptomatic or to evoke mild disease that may persist undetected and result in transmission through blood donations [1,3,5,15–17]. In contrast, the elderly, neonates, splenectomized, immunocompromised, and persons co-infected with other tick-transmitted pathogens are more likely to be symptomatic with malaise, fever, chills, and fatigue, and may also be at increased risk for severe disease-related complications, including but not limited to hemolytic anemia, acute respiratory failure, congestive heart failure, and renal failure, which may result in death [1–5,18,19]. The recommended treatment combinations for babesiosis include atovaquone plus azithromycin or clindamycin plus quinine, with red cell exchange transfusions used for life-threatening infections [1–4,20–22].

Over the past decade, there have been a growing number of reported cases in the United States, including transfusion-transmitted babesiosis (TTB) cases [3,5,9,11,15,23–26]. In response, efforts are being made to mitigate the risk of human babesiosis infections, including: the development of donor screening tests and testing strategies [27–29], the Food and Drug Administration (FDA)-sponsored workshop on TTB in the U.S. [5], creation of the American Association of Blood Banks’ *Babesia* Task Force, addition of babesiosis to the list of nationally notifiable diseases by Centers for Disease Control and Prevention (CDC), and the FDA Blood Products Advisory Committee meeting [23] on the risk of *Babesia* infection. As elderly persons are at a higher risk for clinical babesiosis with severe complications and published literature suggests a disproportionately high blood utilization in this population, the elderly are more likely to be diagnosed with babesiosis and be at an increased risk for TTB [1–3,5,10,11,25, 26,30–34]. The Centers for Medicare & Medicaid Services (CMS) administers Medicare, a national health insurance that provides coverage to virtually all U.S. elderly persons ages 65 and older, and maintains large administrative databases [35–37]. The primary objective of our retrospective population-based study was to assess babesiosis occurrence among elderly Medicare beneficiaries in the United States during 2006–2013, using CMS databases. This study markedly extends a previous investigation by Menis and colleagues [9].

## Materials and Methods

The study utilized the 100% Inpatient, Outpatient, Skilled Nursing Facility (SNF), and Carrier Standard Analytical Files (SAFs) as well as Medicare Enrollment Files for calendar years (CYs) 2006–2013 to assess occurrence of babesiosis among elderly Medicare beneficiaries ages 65 and above in coordination with CMS and within the SafeRx Project. The Analytical Files contain billing information on medical services rendered (e.g., diagnoses, procedures). The diagnoses and procedures are first recorded into medical records by healthcare providers (e.g., physicians), and then coded and submitted to Medicare for billing and reimbursement purposes, thus ending up in the CMS administrative databases. Specifically, the Inpatient, SNF, Outpatient, and Carrier SAFs contain claims data submitted by inpatient hospital providers, SNFs, institutional outpatient (e.g., hospital outpatient departments, rural health clinics), and non-institutional providers (e.g., physicians, physician assistants, nurse practitioners), respectively. The Medicare Enrollment Files contain demographic and enrollment information and help to ascertain Medicare coverage eligibility. The Analytical and Enrollment Files were linked to ascertain babesiosis occurrence rates. The Medicare’s Part D Prescription Drug Event File was used to extract dispensed prescription drug data in order to ascertain medication treatment of babesiosis. To be eligible for the study in a particular year, beneficiaries had to be enrolled in Medicare fee-for-service Parts A (i.e. hospital insurance) and B (i.e. physician insurance) for at least 365 consecutive days prior to and including the latest month of continuous enrollment in that year. Likely incident babesiosis cases were identified based on the first recording of the International Classification of Diseases, 9th Revision, Clinical Modification (ICD-9-CM) diagnosis code for babesiosis (088.82) during the calendar year, with no recorded babesiosis in the preceding 365 days.

The study assessed babesiosis occurrence rates by estimating the number of cases per 100,000 beneficiaries overall, by calendar year, gender, age, race, and state of residence. States with babesiosis rates of <2 per 100,000 were not displayed. The study also assessed 30-day all-cause mortality as well as ascertained the recorded babesiosis diagnoses in different service settings (e.g., hospital inpatient, institutional outpatient, physician offices) and evaluated seasonal occurrence by diagnosis month for CYs 2006–2013. The seasonality assessment was based on the number of babesiosis cases in each month and the number of beneficiaries continuously enrolled in the fee-for-service Medicare (Parts A and B) within the 365 days of each month in the calendar year. The Medicare beneficiaries with babesiosis were assigned an age based on the diagnosis date, and those without babesiosis had age assessed at the beginning of the latest enrollment month in the year. Beneficiaries with babesiosis were excluded from the analyses of subsequent calendar years or diagnosis months. All statistical analyses, including Chi-Squared tests to compare babesiosis rates by gender and race as well as to ascertain babesiosis occurrence trends by calendar year and age, were conducted using SAS version 9.2.

Among the recorded babesiosis cases, our study investigated possible co-infections with two other tick-borne illnesses: Lyme disease and/or Ehrlichiosis. Lyme disease was identified by ICD-9-CM diagnosis code 088.81, whereas Ehrlichiosis was identified by one or more of the following diagnosis codes: 082.40, 082.41, and 082.49. A co-infection was defined as a case in which babesiosis and at least one of these additional diagnoses were recorded on the same claim (i.e. on the same doctor visit or hospital stay). Four mutually exclusive babesiosis groups were evaluated: babesiosis only; babesiosis and Lyme disease; babesiosis and Ehrlichiosis; and babesiosis, Lyme disease, and Ehrlichiosis. For each of these groups, the study investigated prescription drug use in the week (7 days) following the babesiosis diagnosis. As prescription drugs are covered under Medicare Part D, this analysis applied continuous Part D enrollment throughout the 7-day window. Prescription drugs were identified using National Drug Codes (NDC) and stratified into therapeutic categories. Prescription drug utilization was assessed as a percentage of continuously-enrolled cases with at least one NDC recorded.

Our study assessed the frequency of recorded diagnostic tests which can be utilized for detection of babesiosis infection [1,3,30]. Diagnostic tests were identified by Current Procedural Terminology codes recorded on the same claim as the babesiosis diagnosis and were categorized into four types: blood smear, tests to detect antibodies, tests to detect nucleic acids, and the other test (mononuclear cell antigen, quantitative [e.g., flow cytometry]). Utilization of specific diagnostic tests was ascertained as percentage of all babesiosis cases with at least one test recorded on the diagnosis claim, overall and by setting. Our investigation using large CMS databases was granted a categorical exemption by the FDA's IRB, as it uses only existing data and information is recorded in such a manner that subjects cannot be identified.

## Results

**Fig 1:**
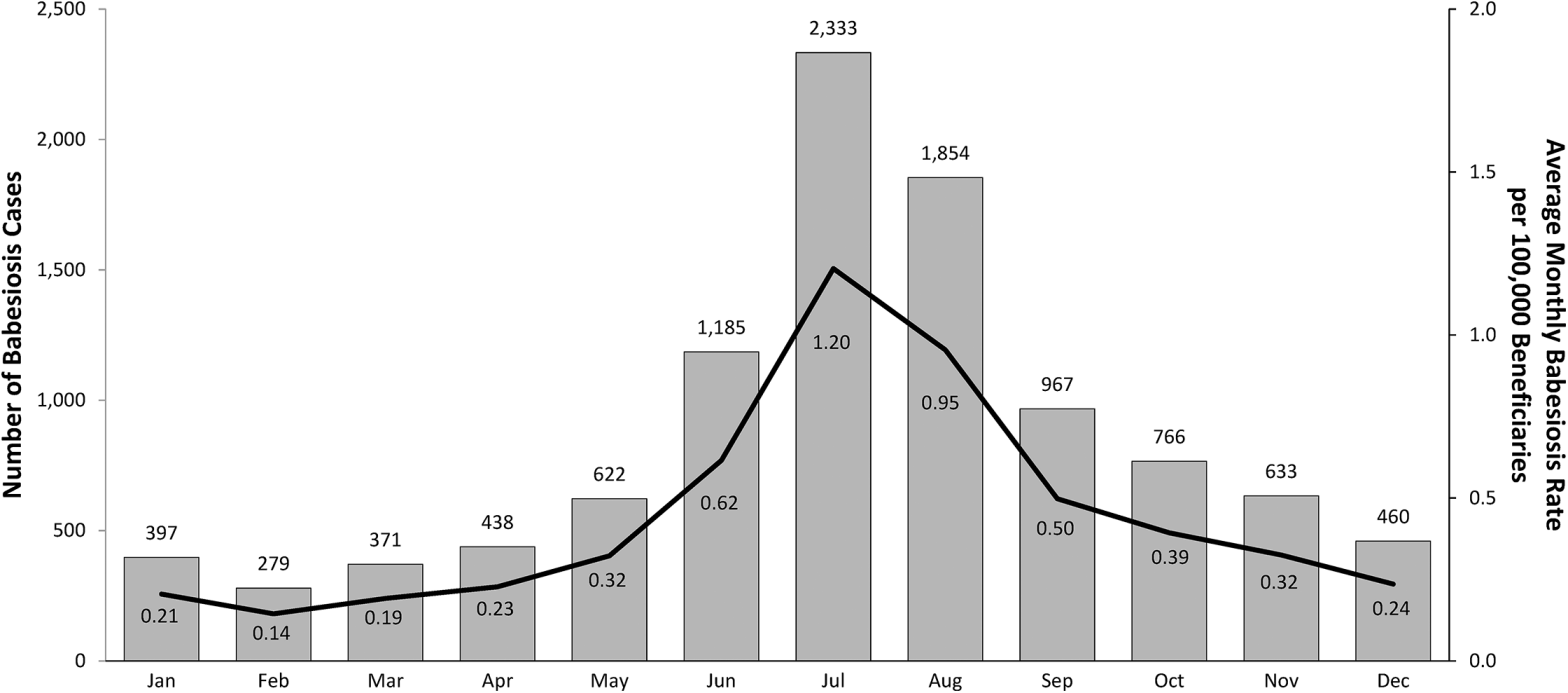
Babesiosis Cases (Gray Bars) and Average Rates (Black Line) by Month of Diagnosis among Elderly Medicare Beneficiaries, 2006–2013.

A total of 10,305 elderly Medicare beneficiaries had a recorded babesiosis diagnosis during 2006–2013 study period, for an overall rate of about 5 per 100,000 persons. Table 1 shows overall and annual number of babesiosis cases with corresponding rates (per 100,000 elderly persons) for all beneficiaries as well as by age and sex. The results showed a significant increase in the national babesiosis occurrence during the eight-year period, overall and by sex, with the lowest number of babesiosis cases in 2007 (N = 852) and the largest number in 2013 (N = 1,848). Table 1 shows a significantly lower risk of babesiosis occurrence with increasing age (p<0.001). About 90% of babesiosis cases were elderly ages 65 to 84; and only 10% of cases were older elderly of 85 and above. The overall babesiosis rates (per 100,000) were significantly higher among males as compared to females, 5.57 vs. 4.48, p<0.001 (RR = 1.24; 95%CI 1.20–1.29); and among whites vs. non-whites, 5.48 and 1.36, respectively, p<0.001 (RR = 4.02; 95%CI 3.62–4.46). Approximately 71% of the babesiosis diagnoses were recorded in physician offices, 11% in institutional outpatient setting, and 18% in hospital inpatient setting. About 60% of 10,305 cases had either Lyme disease and/or Ehrlichiosis recorded on the same claim. About 0.9% (N = 91) of 10,305 cases died within 30 days of being diagnosed. A 30-day mortality rate for babesiosis cases diagnosed in the inpatient setting was 3.3% (59 of 1,804) (data not displayed). Fig 1 shows the number of babesiosis cases and corresponding average rates by diagnosis months during 2006–2013. The results show the summer months of June, July, and August to be the peak months for babesiosis occurrence. About half of all cases (52.1%) were diagnosed in the three summer months, whereas 75% of cases were diagnosed in the months of May through October. (Fig 1)

**Table 1:** Babesiosis Cases and Rates among Elderly Medicare Beneficiaries, Overall, by Sex and Age, During 2006–2013.

	Number of Babesiosis Cases (Babesiosis Rate per 100,000 Beneficiaries^a^)								
Sex and Age	All Years, 2006–2013	2006	2007	2008	2009	2010	2011	2012	2013
All Beneficiaries									
All Ages (65+)^b^	10,305 (5)	994 (4)	852 (3)	1,222 (5)	1,496 (6)	1,308 (5)	1,366 (5)	1,219 (5)	1,848 (7)
65–74	5,754 (6)	536 (4)	439 (4)	676 (6)	832 (7)	731 (6)	755 (6)	716 (6)	1,069 (8)
75–84	3,513 (5)	355 (3)	315 (3)	423 (4)	511 (6)	446 (5)	475 (5)	382 (4)	606 (7)
≥ 85	1,038 (3)	103 (2)	98 (2)	123 (3)	153 (3)	131 (3)	136 (3)	121 (3)	173 (4)
Age Trend P-Value^c^	<0.001	<0.001	<0.001	<0.001	<0.001	<0.001	<0.001	<0.001	<0.001
Females									
All Ages (65+)^b^	5,397 (4)	512 (3)	443 (3)	647 (4)	812 (6)	692 (5)	712 (5)	648 (4)	931 (6)
65–74	3,128 (6)	283 (4)	242 (4)	364 (6)	458 (7)	407 (6)	424 (7)	400 (6)	550 (8)
75–84	1,708 (4)	174 (3)	153 (3)	214 (4)	262 (5)	209 (4)	228 (4)	179 (4)	289 (6)
≥ 85	561 (2)	55 (2)	48 (2)	69 (2)	92 (3)	76 (2)	60 (2)	69 (2)	92 (3)
Age Trend P-Value^c^	<0.001	<0.001	<0.001	<0.001	<0.001	<0.001	<0.001	<0.001	<0.001
Males									
All Ages (65+)^b^	4,908 (6)	482 (4)	409 (4)	575 (5)	684 (6)	616 (6)	654 (6)	571 (5)	917 (8)
65–74	2,626 (6)	253 (4)	197 (4)	312 (6)	374 (7)	324 (6)	331 (6)	316 (5)	519 (9)
75–84	1,805 (6)	181 (4)	162 (4)	209 (5)	249 (6)	237 (6)	247 (7)	203 (5)	317 (8)
≥ 85	477 (4)	48 (4)	50 (4)	54 (4)	61 (4)	55 (4)	76 (5)	52 (3)	81 (5)
Age Trend P-Value^c^	<0.001	0.271	0.479	0.026	0.005	0.048	0.714	0.010	<0.001

^a^ Babesiosis rates are rounded to the nearest whole number.

^b^ The trend of Babesiosis occurrence rates during 2006–2013 is statistically significant according to the Cochran-Armitage test for trend, using a significance level of p<0.05.

^c^ Cochran-Armitage test for trend used to evaluate babesiosis rates across age categories.

Table 2 shows overall and annual number of babesiosis cases with corresponding rates (per 100,000 beneficiaries) by states. The highest overall babesiosis rates (per 100,000) were identified in Connecticut (46), Massachusetts (45), Rhode Island (42), New York (27), and New Jersey (14), with rates of up to 10 times higher than the U.S. national rate. The top five babesiosis-endemic states accounted for 76.5% of all cases identified in the U.S. elderly. Other states also had babesiosis recorded, including but not limited to Maryland (7), Virginia (4), Pennsylvania (3), Florida (3), and California (2), which represented 14.5% of all cases recorded. Significantly increasing babesiosis occurrence trends were identified nationally and in most states displayed, with the highest rates occurring in 2013 overall and for many states, including but not limited to Massachusetts, Connecticut, Rhode Island, and New Jersey. Significantly increasing trends were also found in New Hampshire, Maine, Pennsylvania, Florida, California, and some other states. In contrast, significantly declining babesiosis occurrence trends were found in Maryland and Virginia, with the lowest rates in 2011 and 2013, respectively. (Table 2; the results are also shown in Figures A-R in S1 File)

**Table 2:** Babesiosis Cases and Rates among Elderly Medicare Beneficiaries, Overall and by State, During 2006–2013.

	Number of Babesiosis Cases (Babesiosis Rate per 100,000 Beneficiaries^a^)								
State^b^	All Years, 2006–2013	2006	2007	2008	2009	2010	2011	2012	2013
Total Cases^c^	10,305 (5)	994 (4)	852 (3)	1,222 (5)	1,496 (6)	1,308 (5)	1,366 (5)	1,219 (5)	1,848 (7)
Connecticut^c^	1,307 (46)	173 (44)	166 (44)	183 (50)	156 (44)	122 (35)	153 (45)	128 (38)	226 (67)
Massachusetts^c^	2,161 (45)	76 (13)	128 (22)	185 (32)	229 (39)	230 (39)	350 (59)	391 (63)	572 (89)
Rhode Island^c^	247 (42)	34 (45)	13 (18)	30 (41)	33 (46)	27 (38)	28 (38)	21 (28)	61 (79)
New York	3,193 (27)	397 (24)	269 (17)	381 (25)	595 (41)	535 (37)	334 (23)	253 (18)	429 (30)
New Jersey^c^	980 (14)	80 (9)	59 (7)	98 (11)	150 (17)	112 (13)	169 (19)	127 (14)	185 (21)
Maryland^c^	312 (7)	43 (8)	34 (7)	60 (12)	44 (8)	39 (7)	24 (4)	28 (5)	40 (7)
New Hampshire^c^	85 (7)	3 (2)	4 (3)	9 (6)	6 (4)	10 (7)	20 (13)	21 (13)	12 (7)
Maine^c^	76 (6)	8 (4)	4 (2)	10 (6)	6 (4)	9 (5)	16 (10)	10 (6)	13 (8)
District of Columbia	15 (4)	3 (7)	1 (2)	3 (7)	1 (2)	0 (0)	3 (7)	3 (7)	1 (2)
Virginia^c^	245 (4)	25 (3)	30 (4)	58 (8)	33 (5)	28 (4)	26 (4)	23 (3)	22 (3)
Minnesota^c^	104 (4)	6 (1)	8 (2)	8 (2)	18 (5)	17 (5)	28 (9)	5 (2)	14 (5)
Vermont	24 (4)	2 (3)	2 (3)	3 (4)	3 (4)	1 (1)	7 (9)	2 (2)	4 (5)
Pennsylvania^c^	262 (3)	28 (2)	18 (2)	31 (3)	22 (2)	24 (3)	36 (4)	40 (4)	53 (5)
Delaware	25 (3)	2 (2)	1 (1)	7 (7)	4 (4)	3 (3)	4 (4)	1 (1)	3 (3)
Wisconsin^c^	111 (3)	5 (1)	13 (2)	14 (3)	10 (2)	17 (4)	23 (5)	8 (2)	21 (4)
Florida^c^	393 (3)	29 (2)	33 (2)	44 (2)	70 (4)	50 (3)	53 (3)	59 (3)	55 (3)
California^c^	279 (2)	15 (1)	10 (1)	41 (2)	49 (2)	31 (2)	43 (2)	40 (2)	50 (2)

^a^ Babesiosis rates are rounded to the nearest whole number.

^b^ States include District of Columbia. States are shown in descending order of average babesiosis rate during the 8-year study period.

^c^ The trend of Babesiosis occurrence rates during 2006–2013 is statistically significant according to the Cochran-Armitage test for trend, using a significance level of p<0.05.

Figures S-V in S1 File show diagnostic tests recorded in different settings for Medicare beneficiaries diagnosed with babesiosis. Blood smear test was recorded more frequently than other tests in the institutional settings, while antibody and nucleic acid tests were more commonly recorded in the physician office setting (Figures S-V in S1 File). Table 3 shows recorded medication use within seven days of babesiosis diagnosis in the four mutually exclusive babesiosis groups. About 36% of babesiosis-only cases with any NDC recorded had a CDC-recommended [21] babesiosis treatment combination of atovaquone and azithromycin, and 38% had no recorded medication treatment, as listed in the table. In comparison, only 16% had the same babesiosis treatment combination and 42% had no recorded treatment among persons with both babesiosis and Lyme disease recorded on the same claims. About 32% of babesiosis-only cases had a recorded treatment with tetracyclines as compared to 49% of persons with both babesiosis and Ehrichiosis recorded. Of 10,305 cases, 32 (0.3%) had exchange transfusion within 7 days of diagnosis (data not shown).

**Table 3:** Medication Use in the 7 Days following Babesiosis Diagnosis, Overall and in the Four Babesiosis Groups.

	Number of Babesiosis Cases (Percent of Cases with Any NDC^b^ recorded)				
Medication	All Babesiosis Cases N = 10,305	Babesiosis Only N = 4,353	Babesiosis & Lyme disease N = 2,490	Babesiosis & Ehrlichiosis N = 362	Babesiosis & Lyme disease & Ehrlichiosis N = 3,100
Continuously-Enrolled Cases^a^	4,897	2,066	1,190	157	1,484
Cases with Any NDC^b^	4,526 (100)	1,937 (100)	1,098 (100)	139 (100)	1,352 (100)
All Anti-Infectives	1,149 (25)	809 (42)	239 (22)	40 (29)	61 (5)
Atovaquone	1,037 (23)	758 (39)	212 (19)	35 (25)	32 (2)
Clindamycin	87 (2)	57 (3)	17 (2)	2 (1)	11 (1)
All Anti-Malarials	194 (4)	89 (5)	87 (8)	1 (1)	17 (1)
Hydroxychloroquine Sulfate	86 (2)	22 (1)	50 (5)	1 (1)	13 (1)
Quinine	37 (1)	29 (2)	7 (1)	0 (0)	1 (0)
All Cephalosporins	204 (5)	66 (3)	87 (8)	8 (6)	43 (3)
Cefuroxime Axetil	96 (2)	27 (1)	40 (4)	6 (4)	23 (2)
All Macrolides	1,241 (27)	830 (43)	290 (26)	42 (30)	79 (6)
Azithromycin	1,156 (26)	808 (42)	248 (23)	40 (29)	60 (4)
All Penicillins	248 (6)	85 (4)	75 (7)	10 (7)	78 (6)
Amoxicillin	160 (4)	55 (3)	46 (4)	6 (4)	53 (4)
All Tetracyclines	1,336 (30)	617 (32)	373 (34)	68 (49)	278 (21)
Doxycycline Hyclate	1,229 (27)	571 (30)	323 (29)	64 (46)	271 (20)
NDC Combinations					
Atovaquone + Azithromycin	929 (21)	700 (36)	170 (16)	32 (23)	27 (2)
Clindamycin + Quinine	31 (1)	25 (1)	6 (1)	0 (0)	0 (0)
No Treatment^c^	2,156 (48)	736 (38)	457 (42)	48 (35)	915 (68)

^a^ Cases in which the beneficiary was continuously enrolled in Medicare Part D (prescription drug coverage) during the 7 days following diagnosis.

^b^ Cases with at least one NDC recorded during the 7-day observation window.

^c^ Cases with at least one NDC recorded but who were not treated with any of the medications listed above.

## Discussion

Our population-based study among the U.S. elderly showed a significant increase in the national babesiosis occurrence trends during the eight-year study period. The study identified the highest babesiosis occurrence in babesiosis-endemic states of Connecticut, Massachusetts, Rhode Island, New York, and New Jersey, with rates of up to 10 times higher than the U.S. national rates. A substantial number of recorded *Babesia* cases were also identified in Maryland, New Hampshire, Maine, Virginia, Pennsylvania, Florida, and California, thus suggesting an expansion of *Babesia* infection into non-endemic areas. In addition, significantly increasing babesiosis occurrence trends during the eight-year study period were identified in most states with babesiosis rates of ≥2 per 100,000, with the highest rates occurring in 2013 both nationally and in many endemic states. In contrast, most of the states with babesiosis rates of <2 per 100,000 did not have significant babesiosis occurrence trends during the study period (data not displayed), thus suggesting a possible lack of natural transmission. Babesiosis occurrence was significantly higher for males vs. females and for whites vs. non-whites, with the majority of babesiosis cases diagnosed in the months of May through October. The study suggests that blood smear is more commonly utilized for diagnosis in the institutional settings, whereas antibody and nucleic acid tests are more commonly used in the physician office setting. The results also suggest that about a quarter of cases are diagnosed based on antibody testing alone, which is a strategy that lacks the ability to discriminate between past exposure versus active infection, and needs further confirmation.

The study results on medication treatment of babesiosis suggest a potentially frequent occurrence of inappropriate or lack of treatment, especially among babesiosis cases co-infected with either Ehrlichiosis and/or Lyme disease, which needs further investigation. Specifically, although the recommended treatment for babesiosis includes atovaquone plus azithromycin or clindamycin plus quinine [1–3,20,21], the study identified only about 21% of cases with CDC-recommended babesiosis treatment combinations [21], about 30% of cases with recorded potentially inappropriate tetracycline treatment, and a substantial proportion of cases (about 48%) with no recorded medication treatment. Persons co-infected with either Lyme disease and/or Ehrlichiosis were less likely to have the recommended babesiosis treatment combinations and more likely to have no recorded medication treatment as compared to babesiosis-only group. Additionally, future population-based investigations of unconventional regimens are needed to help further ascertain current practice for treatment of babesiosis.

Overall, our study findings, in concordance with CDC’s national surveillance results, suggest increasing babesiosis occurrence trends over time, with the highest rate in 2013 [11,38]. Although babesiosis has been a notifiable disease since January 2011, babesiosis occurrence as reported by states to CDC [10,11,38] was noticeably lower than the recorded babesiosis occurrence identified in our study, which could be due to under-reporting to CDC[10,11] as well as a higher likelihood of under-diagnosing babesiosis in the general population versus elderly since babesiosis is more likely to be asymptomatic in younger individuals as compared to older persons [3,5,16,17]. Similarly, in support of our study’s results, surveillance in the babesiosis-endemic states also suggests an increasing occurrence of babesiosis over time [39–45]. The findings of lower overall babesiosis occurrence in Wisconsin and Minnesota are supported by the literature and could be due to environmental factors as well as a potentially restricted occurrence of babesiosis to portions of those states [7,27,46]. As supported by the literature [3,5,9,10–12,39,41,47], our study also shows that most of the babesiosis cases among the elderly are diagnosed in the months of May through October, with the peak occurrence in June, July, and August. These findings are likely related to increased activity of humans during summer months as well as the lifecycle and activity of the tick vector and mammalian hosts [1,3–5]. In support of the published data [9–11,13,39–41,47,48], our study further shows that babesiosis occurrence varies by gender and age, with higher rates among males as compared to females and in younger elderly as compared to older elderly persons. These findings could be associated with greater outdoor activity in males vs. females and in younger vs. older persons [49,50]. Also, the study findings of substantially increased risk of human babesiosis infection in whites vs. non-whites could be due to a higher leisure-time physical activity in whites, which needs further investigation [49,51,52]. In support of the literature [18,19,53–55], the study suggests a high co-infection rate with either Lyme disease or Ehrlichiosis for recorded babesiosis cases. Additionally, as most of the cases were diagnosed in the outpatient settings and resulted in a low 30-day mortality, our study, in concordance with the literature, suggests that a majority of babesiosis cases in the U.S. elderly are likely to be mild or moderate [2–5]. In contrast, as supported by the literature [12,13,48], our study identified a substantially higher mortality among babesiosis cases diagnosed in the inpatient setting.

Our study is based on the administrative databases, and consequently, limitations include: difficulty in identifying incident versus prevalent cases due to persistent parasitemia in some cases, possible misdiagnosis or misrecording of babesiosis, diagnostic tests, and medication use as well as lack of clinical detail for diagnosis code verification, identification of TTB cases, and ascertainment of specific *Babesia* species. The claims data also could not distinguish human granulocytic anaplasmosis caused by *Anaplasma phagocytophilum* (previously known as human granulocytic ehrlichiosis) from Ehrlichiosis infections. Since only about 50% of cases had at least one diagnostic test recorded, clinical investigations are needed to identify all diagnostic tests used and verify the validity of the recorded diagnostic testing in claims data. During the study period, there was no FDA-approved test for babesiosis screening in blood donors, although non-validated nucleic acid and antibody-based laboratory tests were available for diagnosis, which may have added a potential uncertainty and variability of the study results [4,5,23,30]. Additionally, although Medicare administrative databases include information on laboratory tests performed, test results are not generally available in claims data. Future epidemiologic investigations will need medical record review to assess positive predictive value of the ICD-9-CM diagnosis code for babesiosis and further support its routine use in monitoring impact of the disease and public health interventions. The potential contribution of increased babesiosis awareness by medical community in non-endemic areas to the trends identified in our study merits further evaluation in appropriate settings. Also, beneficiary travels and shipments of blood components throughout the country may have contributed to the babesiosis occurrence trends identified in non-endemic states. The administrative data utilized do not provide population-based information on babesiosis occurrence among persons under 65 in the United States. However, as suggested by the literature, younger persons are more likely to be asymptomatic and, therefore, less likely to be diagnosed with the disease [3,5,16,17].

Our study is the largest-to-date national population-based investigation of babesiosis occurrence among the U.S. elderly, which shows variations in babesiosis occurrence by year, state of residence, age, gender, race, clinical setting, and diagnosis months. Overall, our eight-year study (2006–2013) identified increasing babesiosis occurrence trends over time, with highest rates in the five babesiosis-endemic states of Connecticut, Massachusetts, Rhode Island, New York, and New Jersey. The study, therefore, suggests the need for prevention strategies, including but not limited to deer population control, enhanced public awareness of tick-transmitted infections, and blood donor testing as well as the need for monitoring for potential expansion of human babesiosis to non-endemic areas. Human encroachment into tick and deer habitat, changes in climate, growth of the deer population, climatic effects on tick populations, and travel to babesiosis-endemic areas may be responsible for the spread of the infection to previously non-endemic states (e.g., Maine, New Hampshire, Pennsylvania) [3–5,56–58]. Moreover, our study suggests the need for greater physician awareness of CDC-recommended babesiosis treatment [21]. As the results are likely to be indicative of babesiosis transmission rates in different geographic areas, our study will assist in determining appropriate blood donor testing strategies (e.g., universal donor testing, regional donor testing) for maximizing prevention of transfusion-transmission and minimizing negative effects on the U.S. blood supply. Since the elderly utilize most of the transfused blood [31–34], future studies are also needed to focus on evaluating the risk of TTB among the elderly. Overall, the results of this study suggest that large administrative databases could play an important role in assessing the occurrence (especially trends) of emerging infections in the United States and abroad as well as in helping to inform policies and evaluate the effectiveness of different risk reduction strategies to the nation’s blood supply.

## Supporting Information

S1 File Supporting Information Figures A-V. (DOC)
