# Supplementary material for: Babesiosis Occurrence among the Elderly in the United States, as Recorded in Large Medicare Databases during 2006–2013
Source: PLoS One. 2015 Oct 15;10(10):e0140332. doi: 10.1371/journal.pone.0140332 (PMC4607449; doi:10.1371/journal.pone.0140332)
Supplement: S1 File — (DOC) [file pone.0140332.s001.doc]

**Supporting Information**

**Figure A. Babesiosis Cases (Gray Bars) and Rates (Black Line) by Year among Elderly Medicare Beneficiaries, United States, 2006-2013**


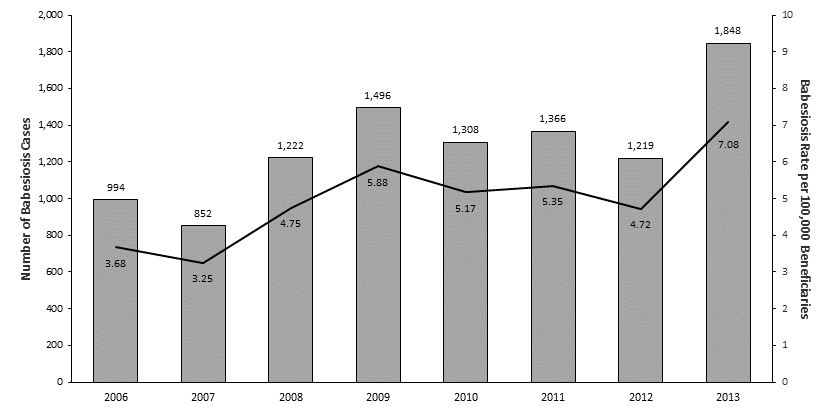


**Figure B. Babesiosis Cases (Gray Bars) and Rates (Black Line) by Year among Elderly Medicare Beneficiaries, Connecticut, 2006-2013**

**Figure C. Babesiosis Cases (Gray Bars) and Rates (Black Line) by Year among Elderly Medicare Beneficiaries, Massachusetts, 2006-2013**

**Figure D. Babesiosis Cases (Gray Bars) and Rates (Black Line) by Year among Elderly Medicare Beneficiaries, Rhode Island, 2006-2013**

**Figure E. Babesiosis Cases (Gray Bars) and Rates (Black Line) by Year among Elderly Medicare Beneficiaries, New York, 2006-2013**

**Figure F. Babesiosis Cases (Gray Bars) and Rates (Black Line) by Year among Elderly Medicare Beneficiaries, New Jersey, 2006-2013**

**Figure G. Babesiosis Cases (Gray Bars) and Rates (Black Line) by Year among Elderly Medicare Beneficiaries, Maryland, 2006-2013**

**Figure H. Babesiosis Cases (Gray Bars) and Rates (Black Line) by Year among Elderly Medicare Beneficiaries, New Hampshire, 2006-2013**

**Figure I. Babesiosis Cases (Gray Bars) and Rates (Black Line) by Year among Elderly Medicare Beneficiaries, Maine, 2006-2013**

**Figure J. Babesiosis Cases (Gray Bars) and Rates (Black Line) by Year among Elderly Medicare Beneficiaries, Washington DC, 2006-2013**

**Figure K. Babesiosis Cases (Gray Bars) and Rates (Black Line) by Year among Elderly Medicare Beneficiaries, Virginia, 2006-2013**

**Figure L. Babesiosis Cases (Gray Bars) and Rates (Black Line) by Year among Elderly Medicare Beneficiaries, Minnesota, 2006-2013**

**Figure M. Babesiosis Cases (Gray Bars) and Rates (Black Line) by Year among Elderly Medicare Beneficiaries, Vermont, 2006-2013**

**Figure N. Babesiosis Cases (Gray Bars) and Rates (Black Line) by Year among Elderly Medicare Beneficiaries, Pennsylvania, 2006-2013**

**Figure O. Babesiosis Cases (Gray Bars) and Rates (Black Line) by Year among Elderly Medicare Beneficiaries, Delaware, 2006-2013**

**Figure P. Babesiosis Cases (Gray Bars) and Rates (Black Line) by Year among Elderly Medicare Beneficiaries, Wisconsin, 2006-2013**

**Figure Q. Babesiosis Cases (Gray Bars) and Rates (Black Line) by Year among Elderly Medicare Beneficiaries, Florida, 2006-2013**

**Figure R. Babesiosis Cases (Gray Bars) and Rates (Black Line) by Year among Elderly Medicare Beneficiaries, California, 2006-2013**

**Figure S. Diagnostic Tests Recorded on the Same Claim as Incident Babesiosis Cases, All File Settings**

**Figure T. Diagnostic Tests Recorded on the Same Claim as Incident Babesiosis Cases, Inpatient Setting**

**Figure U. Diagnostic Tests Recorded on the Same Claim as Incident Babesiosis Cases, Institutional Outpatient Setting**

**Figure V. Diagnostic Tests Recorded on the Same Claim as Incident Babesiosis Cases, Physician Office Setting**
